# Supplementary material for: Population structure of community-acquired extended-spectrum beta-lactamase producing Escherichia coli and methicillin resistant Staphylococcus aureus in a French region showed no difference between urban and rural areas
Source: PLoS One. 2023 Nov 16;18(11):e0294433. doi: 10.1371/journal.pone.0294433 (PMC10653544; doi:10.1371/journal.pone.0294433)
Supplement: Attachment — Submitted filename: Response to Reviewers.docx [file pone.0294433.s003.docx]

**Reviewer #1:**

Antimicrobial resistance is an urgent global public health threat, with high mortality rates due to infections caused by multidrug-resistant bacteria. Understanding the molecular epidemiology of multidrug-resistant bacteria acquired in the community is crucial for effectively preventing and controlling infections caused by these resistant strains.

There are also some major concerns,

1. **In line 74, the authors mentioned conducting a prospective multicenter study. It is unclear whether this study underwent ethical review. Moreover, the sampling methods and detailed information regarding the samples have not been specified.**

The study was approved by the local ethic committee before the study commencement considering that no patients data were collected and samples were anonymised. This was added in the text line 74. Information regarding samples were specified in Lines 77-80.

2. **In Figure 1, it would be helpful to annotate each circle with specific information to indicate what each circle represents.**

The colors are listed in the figure legend from the inner circle to the outer circle.

3. **Do the authors analyze the MLST of the MRSA isolates?**

We chose to analyse complex clones (CC) rather than sequence types (ST) due to the small size of our population. The results of the MLST analysis are available in the supplementary data.

4. **Do the authors compare the evolutionary changes of multidrug-resistant bacteria between 2019 and 2021 in the region?**

We observed no difference between 2019 and 2021 for phylogroups (p = 0.36), sequence type (p = 0.153) and for the ESBL gene (p = 0.092) in our *Escherichia coli* strains. For *Staphylococcus aureus*, we observed no difference for the complex clonal (p = 0.107) or the SCCmec cassette (p = 0.096). This was added in the text line 135 and line 204.

**Reviewer #2:**

Biguenet et al. has performed a population structure/genomic analysis of ESBL producing E. coli and MRSA isolates in France - reporting the prevalence of STs and AMR genes. The authors has also studied the distribution of these characteristics in relation to the region/areas based on population size. In general the study was interesting and important to public health, and the MS was well written. However, the classification of three areas such as urban and rural was quite arbitrary, and not convincing.

Major questions:

1. **Line 79: population size was not the only factor. other factors such as anthropogenic activities, animal farming, agriculture, or winery would be more relevant to the relationship of isolates under one health approach. So the authors did not know where the patients were from?**

No patient data were collected, so we agree with the reviewer that the location of the laboratory is a distant proxy of patient environment and anthropogenic activities. This limitation is addressed in the discussion section.

2. **Line 169-170: The association of ESBLs and plasmids was not based on solid methodology, as only short-read sequencing data were used to determine the genetic location of these genes. Multicopy insertion elements may fragment the short-read assemblies into multiple contigs, and reconstruction of ESBL plasmids requires additional experiments, such as long-read sequencing or PCR gap closure.**

We agree that short reads do not allow total plasmid reconstruction. We have used PlaScope here, a tool to predict whether the contigs from short read data in our fasta file are from chromosome, plasmid or unclassified location. PlaScope uses Centrifuge and a custom database of chromosome and plasmid sequences to classify the contigs. PlasScope is not able to reconstruct the plasmid or predict if 2 contigs are from the same or different plasmids.

Other tools are also available to distinguish between plasmid and short read contigs:
- Robertson J and Nash J. RFPlasmid: predicting plasmid sequences from short-read
data using machine learning. Microbial Genomics. 2018. DOI: 10.1099/mgen.0.000683
- van der Graaf-van Bloois et al. MOB-suite: software tools for clustering, reconstruction and typing of plasmids from draft assemblies. Microbial Genomics. 2021. DOI: 10.1099/mgen.0.000206 (nécessaire ces 2 citations ?)

3. **Line 298-304: The lack of clinical data or relevant metadata had weakened the discussion and entire investigation. It was unusual that the origin of these isolates were unknown. Without knowing the source, all the discussion was kind of speculation, even though Ec ST131 tended to be circulated among the communities. Was the study supported by the ethical board?**

We agree with the reviewer that the lack of clinical data is the major limitation of our study and we underlined this point in the discussion. However, we have collected all consecutive ESBLE and MRSA isolates in a large number of private community laboratories, and it is very likely that our results reflect population structures of ESBLE and MRSA in our region. Moreover, such data are not frequently available in the community sector.

The study was approved by the local ethic committee before the study commencement considering that no patients data were collected and samples were anonymised
